# Solitary fibrous tumor of the lung: a case report

**DOI:** 10.1186/s40792-016-0286-7

**Published:** 2017-01-07

**Authors:** Yoshinobu Ichiki, Keisei Kakizoe, Takayuki Hamatsu, Atsuji Matsuyama, Taketoshi Suehiro, Fumihiro Tanaka, Masanori Hisaoka, Keizo Sugimachi

**Affiliations:** 1 Department of Chest Surgery, Onga Nakama Medical Association Onga Hospital, 1725-2 Ooaza-Ozaki Ongacho, Onga-gun, Fukuoka, 811-4342 Japan; 2 Department of Surgery, Onga Nakama Medical Association Onga Hospital, Onga-gun, Fukuoka, Japan; 3 Department of Pathology and Oncology, University of Occupational and Environmental Health, School of Medicine, Kitakyushu, Japan; 4 Department of Emergency, Onga Nakama Medical Association Onga Hospital, Onga-gun, Fukuoka, Japan; 5 Second Department of Surgery, University of Occupational and Environmental Health, School of Medicine, Kitakyushu, Japan

**Keywords:** Lung tumor, Solitary fibrous tumor, Surgery, VATS

## Abstract

Solitary fibrous tumors (SFTs) are relatively rare neoplasms that commonly occur in the pleura. The pathological feature of SFTs is a proliferation of spindle-shaped cells in interlacing or storiform fascicles. SFTs appear to derived from pluripotential submesothelial cells, but not the covering mesothelium. SFTs distinctively show diffuse staining for CD34 but lack staining for smooth muscle markers. We herein report a relatively rare case of a 68-year-old male patient without symptoms, who underwent resection for what was considered to be SFT.

## Background

Solitary fibrous tumors (SFTs) are relatively rare neoplasms that commonly occur in the pleura. They account for <5% of all pleural tumors [1]. Most SFTs are accidental findings on chest radiography. The designation is used for lesions that show a proliferation of spindle-shaped cells in interlacing or storiform fascicles. The tumor is characterized by the tumoral differentiations of pluripotential submesothelial cells, the etiology of which is unknown. SFT was first described by Klemperer and Robin in 1931 [2] and has been successively referred to as localized mesothelioma, localized fibrous tumor, fibrous mesothelioma or pleural fibroma, due to controversies regarding its histogenesis. The term “localized mesothelioma” should be avoided as a synonym for SFT, as cases of true localized mesothelioma with a good prognosis have now been described. We herein report a rare case of a patient with a SFT of the visceral pleura.

## Case presentation

**Fig. 1:**
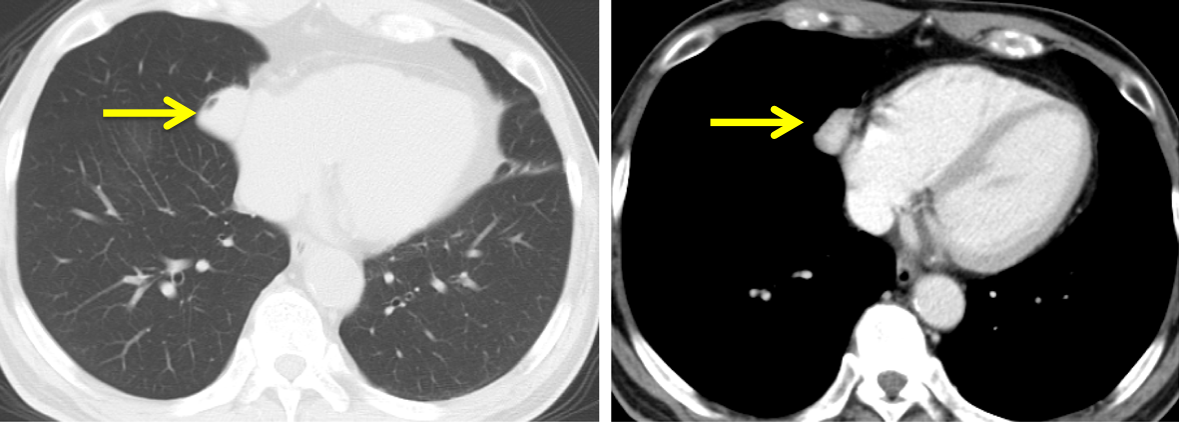
Chest computed tomography (CT) revealed the presence of a well-demarcated solid nodule of 2.6 cm in diameter that was enhanced on contrast-enhanced CT, in the cardio-phrenic angle

The patient was a 68-year-old asymptomatic female who visited our hospital after an abnormal shadow was detected on a chest X-ray. She had no history of occupational exposure to silica, beryllium, or asbestos. Chest computed tomography (CT) revealed the presence of a well-demarcated solid nodule of 2.6 cm in diameter that was enhanced on contrast-enhanced CT, in the cardio-phrenic angle (Fig. 1). We suspected it to be a mediastinal nodule, such as thymoma. A systemic CT examination revealed no tumors other than this tumor.

**Fig. 2:**
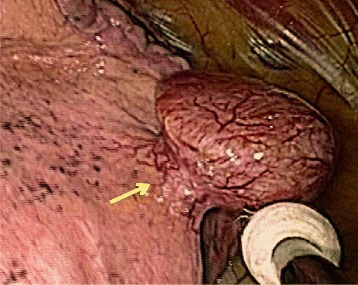
Operative findings showed that a pedunculated tumor rose from the visceral pleura of the *right middle lobe*. A linear stapling device was used to resect the lung nodule. We performed a wedge resection of the *right middle lobe* by video-assisted thoracoscopic surgery (VATS).

**Fig. 3:**
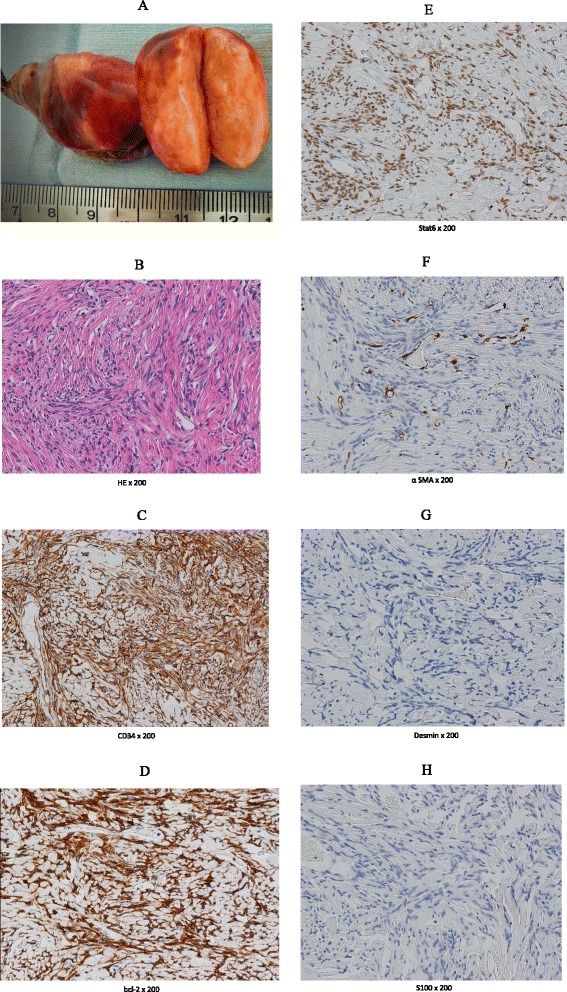
**a** The nodule was firm and solid. On sectioning, it appeared well-demarcated and was yellow-white in color. **b**. The histopathological findings revealed a well-demarcated nodular lesion was composed of a proliferation of spindle or oval cells, admixed with some pleomorphic cells, arranged in a fascicular fashion with ropey collagen fibers, associated with variably dilated blood vessels often displaying staghorn-like appearance, involving the pleural tissue and the pulmonary alveolar tissue. Mitotic figures were few (×200). **c**. Immunohistochemically, the tumor cells were positive for CD34 (×200). **d**. Immunohistochemically, the tumor cells were positive for bcl-2 (×200). **e**. Immunohistochemically, the tumor cells were positive for STAT6 (×200). **f**. Immunohistochemically, the tumor cells were negative for alpha-SMA (×200). **g**. Immunohistochemically, the tumor cells were positive for desmin (×200). **h**. Immunohistochemically, the tumor cells were positive for S-100 (×200).

We decided the nodule was a candidate for surgery to make a diagnosis and cure it. Three ports were placed in the left lateral decubitus position. The lung was deflated to confirm the nodule. Operative findings showed that a pedunculated tumor rose from the visceral pleura of the right middle lobe (Fig. 2). A linear stapling device was used to resect the lung nodule. We performed a wedge resection of the right middle lobe by video-assisted thoracoscopic surgery (VATS). The nodule was firm and solid. On sectioning, it appeared well-demarcated, and was yellow-white in color (Fig. 3a). The macroscopic findings suggested that it was SFT. The postoperative course was uncomplicated. The histopathological findings revealed a well-demarcated nodular lesion was composed of a proliferation of spindle or oval cells, admixed with some pleomorphic cells, arranged in a fascicular fashion with ropey collagen fibers, associated with variably dilated blood vessels often displaying staghorn-like appearance, involving the pleural tissue and the pulmonary alveolar tissue. Mitotic figures were few (Fig. 3b). Immunohistochemically, the tumor cells were positive for CD34 (Fig. 3c), bcl-2 and STAT6, whereas alpha-SMA, desmin, and S-100 were negative. There has been no recurrence in the 11 months since the surgery, and the patient’s condition has remained good.

## Discussion

SFTs of the pleura are relatively rare neoplasms, with an incidence of < 3 per 100000 hospital patients and less than 1000 cases described in the literature. They account for < 5% of all pleural tumors [1]. SFT of the pleura occurs mainly in adults. The sex incidence is equal and they are seen in all ages groups, although they most commonly present in the 60s and 70s. The clinical features depend on the sites, size, and malignant potential of the tumor [3]. The etiological factors are unknown, and asbestos exposure is not correlated with the SFT pathogenesis.

SFT usually presents as a solitary, localized mass with a smooth surface, and glistening capsule attached to the pleura with a richly vascularized pedicle. Some 60 to 80% of SFTs occur from the visceral pleura [4], often in the inferior hemithorax, with a slight predominance of pedunculated tumors [5, 6]. SFTs larger than 10 cm in diameter are usually malignant. In this case, the SFT lesion, showed a solid nodule of 2.6 cm in diameter that was wholly enhanced on contrast-enhanced CT, and no tumors other than this tumor were detected. Therefore, the lesion was likely to be benign SFT based on the imaging findings. They are firm with white-to-gray, whorled cut surfaces [5, 7].

Histologically, SFT is characterized by a multiplicity of growth patterns. The spectrum of histological patterns of SFT of the pleura was clearly illustrated by Moran et al. [8]. In their article, two major histological growth patterns were described: (i) solid spindle and (ii) diffuse sclerosing. Those are admixed in varying proportions. The solid spindle patterns are characterized by variable histological appearances from area to area within the tumor. The short storiform (patternless) is the most frequent pattern in the solid spindle areas and characterized by an oval-to-spindle patterns showing variable short storiform of cartwheel formations. The second most common pattern in the solid spindle areas is hemangiopericytoma-like growth (staghorn). On admission, fibrosarcoma-like, monophasic synovial sarcoma-like, or neural tumor-like patterns can also present focally in the solid spindle areas. The stromal fibrous pattern is more predominant than cellular elements in a diffuse sclerosis pattern. The area of collagenization varies from extensive, diffusely hyalinized, virtually acellular areas, to areas in which scattered cellular elements can still be easily identified. The lesion in this case was pathologically also composed of both proliferation patterns of solid spindle and diffuse sclerosing.

Nuclear overlapping, cellular pleomorphism and tumor necrosis of numerous mitotic figures (>4 per 10 high-power fields) are characteristics of aggressive clinical behavior [5, 6, 9]. Because the mitotic figures were few in this case, it was indicated that the lesion was likely to be benign. It has been reported that 59% of patients with SFT had at least one clinicopathological feature related to malignancy. The mortality and recurrence rates are only 10.2 and 18.2%, respectively [9]. Immunohistochemically, nearly 96% of SFTs are CD34-positive, while the bcl2 and CD99 positivity rates are 94 and 88%, respectively [9]. Calretinin, S-100, desmin, actin-smooth-muscle, actin, neurofilament protein, and EMA are usually negative in SFTs (Table 1) [9–13]. Loss of both CD34 and cytokeratin expression appears to characterize the malignant form of SFT. Usually cytokeratin positivity is very focal; however, in one case of recurrent tumor, 70% of the malignant tumor cells strongly expressed CK AE1/AE3, and a few elements reacted with CAM5.2 [11, 14, 15]. In this case, the immunohistochemical findings were compatible with SFT, as the tumor cells were positive for CD34 and bcl-2, whereas alpha-SMA, desmin, and S-100 were negative.

**Table 1:** Immunohistochemical studies of solitary fibrous tumor of the pleura

Author (Reference)	CD34	bcl-2	Stat6	α SMA	Desmin	S-100
Hanau and Miettinen 1995 [10]	25/28	ND	ND	0/25	3/25	ND
Yokoi et al*.* 1998 [11]	10/10	ND	ND	1/10	1/10	1/10
Chang et al*.* 1999 [12]	13/13	ND	ND	1/13	0/13	0/13
Schirosi et al*.* 2008 [9]	85/88	83/88	ND	ND	ND	ND
Zhu et. al. 2013 [13]	10/12	9/12	ND	ND	ND	0/12

Numbers of cases stained for each marker are shown

*ND* not done

## Conclusions

We herein reported a rare resected case of an SFT of the lung and discussed its differential diagnosis and histogenesis. Early resection is essential, and careful long-term follow-up is needed for the early detection of growth of the residual tumors, as SFT can develop malignant behavior.

## Abbreviations

CT: Computed tomography
SFT: Solitary fibrous tumor
VATS: Video-assisted thoracoscopic surgery
